# Splenic infarction associated with bacterial endocarditis and aortic valve vegetations

**DOI:** 10.3402/jchimp.v2i3.19299

**Published:** 2012-10-15

**Authors:** Muhamed Jasarevic, Christopher Laird, David M. Widlus

**Affiliations:** 1 Department of Medicine, MedStar Union Memorial Hosptial, Baltimore, MD, USA; 2 University of Maryland School of Medicine, Baltimore, MD, USA; 3 Department of Radiology, MedStar Union Memorial Hospital, University of Maryland School of Medicine, Baltimore, MD, USA

Of patients with left-sided endocarditis 20–47% have septic embolization to the spleen with varying degrees of clinical presentation. The natural history varies based on the size and location of the infarct. Larger and more peripheral infarctions are more prone to form abscesses and cause splenic rupture, whereas smaller infarcts may go largely unnoticed and only be detected on incidental imaging. Splenic abscesses are felt due to embolized septic material or secondary infection in a patient with bacteremia.

**Fig. 1:**
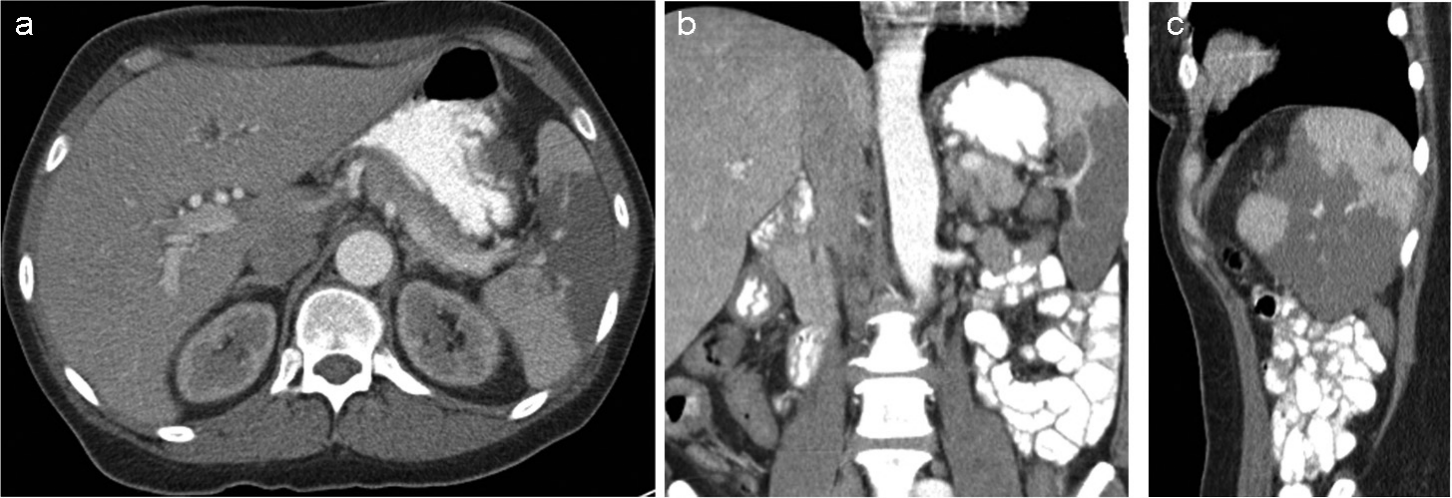
Three views (axial, coronal, and sagittal) from the contrast-enhanced computed tomography scan of the abdomen demonstrate the large wedge-shaped region of decreased radiodensity within the spleen representing the area of infarction.

**Fig. 2:**
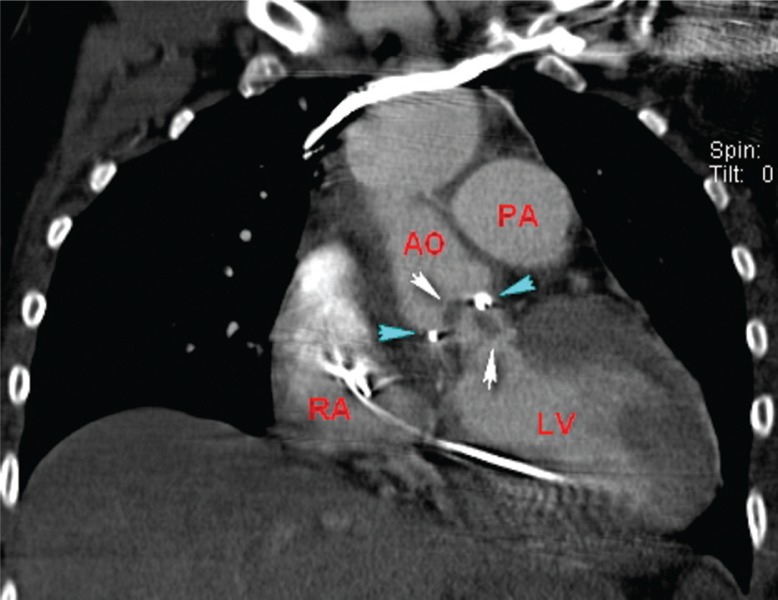
Coronal view from a contrast-enhanced thoracic computed tomography scan demonstrates the extensive vegetations (white arrows) on the aortic valve prosthesis (blue arrows). The right atrium (RA), left ventricle (LV), ascending aorta (AO), and main pulmonary artery (PA) are noted.

The patient in this case is a 45-year-old female with a history of substance abuse, end-stage renal disease on dialysis, and aortic endocarditis status post bioprosthetic valve replacement. The patient presented to the emergency department with a 1-day history of 10/10 left upper quadrant abdominal pain. A computed tomography (CT) scan of the abdomen revealed a large splenic infarct (Fig. 1). A subsequent chest CT and transesophageal echocardiogram demonstrated a large vegetation on the aortic valve with thickening of the valve leaflets (Fig. 2; Movie 1). Accordingly, the patient was treated for bacterial endocarditis and septic embolization to the spleen with infarction.

**Movie 1:**
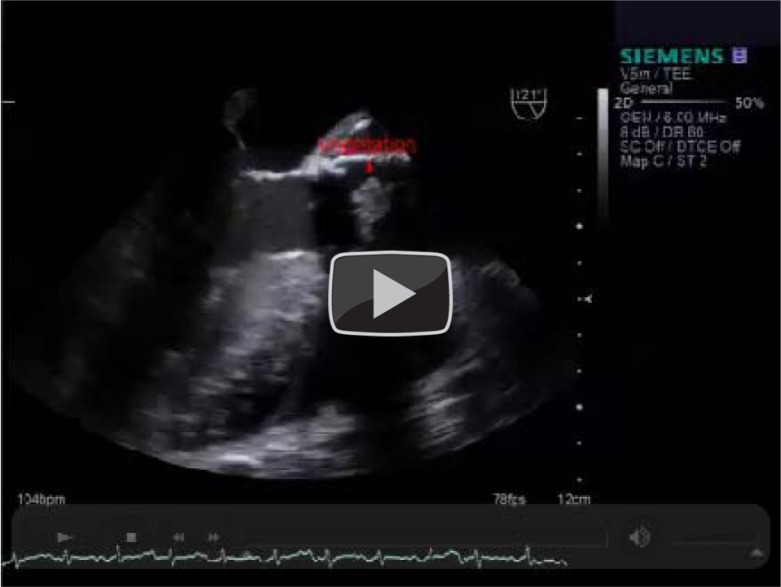
The vegetation can be seen on the valve prosthesis (arrow).

The CT scan appearance of splenic infarction includes a typically triangular region of low radiodensity on portal phase images, following intravenous contrast administration which does not show enhancement on the delayed images. Splenic abscess is strongly suspected if there is gas within the hypodense region. Additional signs of bulging of the splenic capsule, a focal fluid collection, or perisplenic inflammatory stranding are non-specific and each can be seen with bland infarction. Cardiac technique CT scan has shown excellent sensitivity and specificity for detecting aortic valve vegetations as well as for complications such as adjacent abscess or pseudoaneurysm. Pre-operative scanning has been recommended for those who need valve surgery.
